# A Systematically Enhanced LAMP Chip for Rapid, Sensitive, and Contamination-Free Pathogen Detection

**DOI:** 10.1021/acsmeasuresciau.5c00155

**Published:** 2025-12-25

**Authors:** Sajid Uchayash, Jinping Zhao, Md Iqbal Kabir, Junqi Song, Long Que

**Affiliations:** † Department of Electrical and Computer Engineering, 1177 Iowa State University, Ames, Iowa 50011, United States; ‡ Texas A&M AgriLife Research Center at Dallas, Texas A&M University System, Dallas, Texas 75252, United States; § Department of Plant Pathology & Microbiology, Texas A&M University, College Station, Texas 75252, United States

**Keywords:** Isothermal DNA amplification, nanopore thin film sensor, primer immobilization, *Phytophthora infestans*, carryover contamination, pathogen detection

## Abstract

Main techniques for pathogen detection include molecular or nucleic acid–based methods, such as polymerase chain reaction (PCR) and loop-mediated isothermal amplification (LAMP). Among these, chip-based LAMP provides a particularly promising platform for point-of-care diagnostics. To ensure accurate, reliable, and robust pathogen detection, systematic optimization of the LAMP-chip system is essential. Building on our previously developed LAMP-chip platform, we report a systematically advanced version for the rapid and sensitive detection of *Phytophthora infestans*. By systematically investigating the effects of primer concentrations, immobilization ratios, and reaction conditions, we identified an optimal configuration involving immobilized forward inner primers (FIP) on the sensor surface and unmodified backward inner primers (BIP) in solution. This approach enabled the detection of *P. infestans* DNA at concentrations as low as 1 fg/μL, with a transducing optical signal shift of up to 4.33 nm after a 30 min reaction. Further refinement reduced detection time to under 20 min, a 33% reduction of conventional LAMP detection time, without compromising sensitivity. Notably, the use of EDC-NHS chemistry for primer immobilization on the anodic aluminum oxide (AAO) nanopore surface of the LAMP chip effectively minimizes carryover contamination by strictly confining amplification to the chip, representing a major advance over conventional LAMP approaches. This robust, label-free, and user-friendly system offers a promising solution for point-of-care plant pathogen diagnostics, enabling accurate and rapid field-based detection to support timely disease management and improve agricultural outcomes.

## Graphical abstract

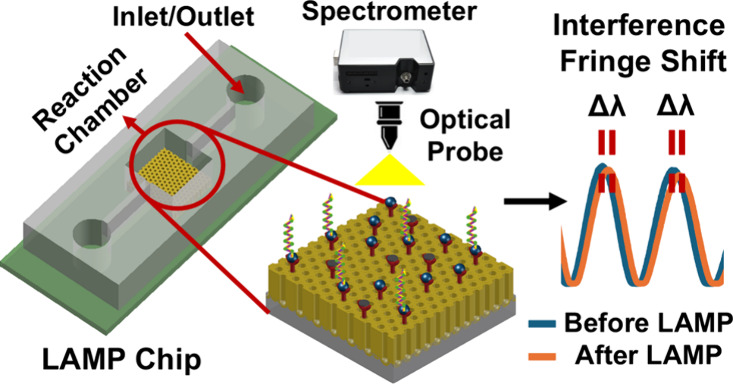

## 1 Introduction

Plant diseases impose a substantial and recurring economic burden on U.S. agriculture, with direct yield losses representing the most significant cost. According to USDA estimates, plant diseases can reduce the potential yield of major crops by 10% to 30% or more, depending on the crop and the scale of an outbreak.^1–3^ In addition to yielding losses, control and management expenses add further financial strain. Farmers spend billions annually on fungicides, bactericides, and other chemical or biological agents, along with the associated labor and equipment required for their application. Trade and market impacts also pose serious challenges. Disease outbreaks can trigger quarantines and export restrictions that severely affect agricultural industries. A notable example is citrus greening disease,^4^ which has caused billions of dollars in revenue losses for Florida’s citrus industry and significantly increased production costs. To address these challenges, continued investment is essential in research and infrastructure including the development of disease-resistant crop varieties, surveillance for emerging pathogens, and strategies for rapid outbreak response.

Point-of-care diagnostics for plant diseases are vital for agriculture, the food industry, and public health.^5–9^ They enable early and rapid detection, which facilitates timely treatment, reduces costs, minimizes crop loss, and supports better disease surveillance for epidemic forecasting and management. Isothermal DNA amplification techniques^10–12^ such as Recombinase Polymerase Amplification (RPA) and Loop-Mediated Isothermal Amplification (LAMP) are powerful molecular tools that detect a pathogen’s genetic material (DNA or RNA) without the need for a thermocycler. These methods operate at a constant temperature, making them suitable for field use with simple, portable devices like battery-powered heat blocks. Detection approaches for these techniques include colorimetric, fluorescent, and lateral flow assays (LFAs).^13–16^ However, these methods are often associated with relatively high detection limits and may require specialized chemicals or equipment such as probes or Raman spectroscopy for accurate on-site or laboratory-based analysis. Integrating CRISPR-Cas systems has significantly improved the specificity and sensitivity of detection, but this also adds to the overall cost and complexity of diagnostics and reagent storage.^17,18^

Despite their many advantages, nucleic acid amplification techniques such as polymerase chain reaction (PCR) and LAMP are highly susceptible to carryover contamination,^19^ which can lead to false-positive results and undermine the accuracy and reliability of both diagnostic and research outcomes. Carryover contamination arises when amplified DNA (amplicons) from a previous reaction inadvertently contaminates subsequent reactions. Given the exponential nature of amplification in both PCR and LAMP, even minute amounts of residual DNA can generate significant and misleading signals. To address this issue, various strategies have been explored.^20,21^ For instance, a Cod-uracil-DNA-glycosylase-based real-time reverse transcriptase LAMP assay (Cod-UNG-rRT-LAMP) was developed specifically to eliminate carryover contamination.^22^ Other studies have demonstrated the effectiveness of incorporating uracil DNA glycosylase (UDG), deoxyuridine triphosphate (dUTP), or mineral oil overlays in LAMP assays to prevent carryover contamination.^23–26^ These approaches have been applied in diverse contexts, including the detection of human malaria,^23^ identification of bacteria in urine samples,^24^ colorimetric detection of SARS-CoV-2,^25^ and identification of foodborne pathogens.^26^ However, they can be limited by incomplete removal of residual DNA, increased assay complexity, potential interference with amplification efficiency, and higher cost or reagent requirements.

Solid-state nanopore film sensors can be functionalized with complex biological molecules, creating a robust and selective sensing platform suitable for detecting proteins, small molecules, and other analytes.^27–29^ The surfaces of the nanopore film sensors also support the immobilization of biomolecules derived from plant pathogens. The transducing signals generated by these sensors include enzymatic electrical signals, fluorescence, and optical interferometry.^30–34^ Recently, we have developed an integrated LAMP-chip system that enables direct solid-phase amplification of plant pathogen DNA using primers covalently attached to the surface of an anodic aluminum oxide (AAO) nanopore thin film.^35^ This platform provides highly sensitive, label-free, and probe-free optical detection. The specificity of the assay is ensured through the use of carefully designed LAMP primers that selectively target unique genetic regions of the pathogen. To the best of our knowledge, this is the first system reported to perform solid-phase LAMP amplification directly on a nanopore thin-film sensor.

In this study, we present an advanced LAMP-chip platform for solid-phase DNA amplification and detection, specifically engineered to maximize sensitivity, reduce assay time, and inherently prevent carryover contamination, using *Phytophthora infestans* strain US-23 as a model pathogen to improve the sensor for broader applications.^36^ A central innovation of this work is the systematic optimization of key parameters, including primer immobilization amounts, primer ratios, and LAMP reaction conditions, which collectively contribute to a highly efficient and user-friendly system. Most importantly, the LAMP-chip platform inherently prevents carryover contamination by confining amplification to primers attached to the chip sensing surface, overcoming a major limitation of conventional LAMP assays. This design enhances assay reliability and makes the platform suitable for both laboratory and on-site applications. As a result, the platform offers a robust, ready-to-use solution for rapid, accurate, and contamination-free pathogen detection in agricultural environments.

## 2 Materials and Methods

### 2.1 Chemicals and Materials

For chip fabrication: Aluminum targets of high purity (99.99%) were purchased from Kurt J. Lesker, Inc. Deionized (DI) water was produced from tap water using a DI water purification system (Millipore, USA). Titanium pellets for electron beam evaporation were purchased from Kurt J. Lesker (Jefferson Hill, PA, USA). Gold sputtering target was purchased from Denton Vacuum, LLC (Moorestown, NJ, USA). Glass substrates (75 mm × 50 mm) were collected from Corning, Inc. (Corning, NY, USA). Polydimethylsiloxane (PDMS) and curing agents (SYLGARD 184 Silicon Elastomer Kit, 2-part) were obtained from Dow Corning (Midland, MI, USA).

Chemicals for sensing surface functionalization: 11-Mercaptoundecanoic acid (HSC_10_COOH, 99%), 8-mercapto-1-Octanol (HSC_8_OH, 98%), N-(3-(Dimethylamino)­propyl)-N’-ethylcarbodiimide hydrochloride (EDC), *N*-hydroxysuccinimide (NHS), and ethanolamine (EA) were purchased from Sigma.

### 2.2 Fabrication Process of the Chip

The fabrication process, as previously described,^37^ began with a glass substrate that underwent a thorough cleaning procedure. The substrate was sonicated sequentially in deionized (DI) water, acetone, ethanol, and finally DI water. Between each step, it was rinsed with DI water and dried using a nitrogen gun. A thin adhesion layer of titanium (∼2 nm) was first deposited on the cleaned glass, followed by the deposition of an aluminum layer (∼2 μm) using electron-beam (e-beam) evaporation. Next, the aluminum layer underwent anodization to form anodic aluminum oxide (AAO) nanopores. Following anodization, another ∼ 2 nm titanium adhesion layer was deposited on top of the masked glass using a Denton e-beam evaporation system. This was followed by the deposition of approximately 5 nm of gold using a Denton Desk IV sputtering system. At this stage, the sensors were ready for experimental use.

### 2.3 Plant and Pathogen Materials

The potato variety Katahdin was maintained as in vitro plants in a growth room. The *Phytophthora infestans* strain US-23 was cultured on rye A medium at 17 °C and maintained in an incubator. For *P. infestans* inoculation, in vitro-cultured potatoe plants were transplanted into soil and grown in a Growtainer at 23 °C under a 16 h/8 h light/dark cycle. Freshly cultured *P. infestans* mycelial mats measuring 1 mm2 were placed upside down on potato leaf laminae two weeks post-transplantation. Infected potato tissues were collected 1-2 weeks post-inoculation.

### 2.4 Chemical Functionalization of the LAMP Sensor Surface

Using a surface functionalization approach similar to that described in our previous work,^35^ 5′-amino-C12-modified inner primers (FIP and BIP) were covalently immobilized on the nanopore sensor surface via EDC/NHS coupling chemistry, as schematically illustrated in Figure 1. Briefly, the sensor surface was first treated with a 0.1 mM mixed solution of HSC_10_COOH and HSC_8_OH (1:9 ratio) and incubated for 30 min, followed by thorough rinsing with ethanol. After air drying, a solution of 0.2 M NHS and 0.05 M EDC was applied to activate surface carboxyl groups and incubated for another 30 min, followed by a PBS rinse. The activated sensor was then immersed in a solution containing 10 μM (or as specified) of 5′-amino-C12-modified FIP and BIP primers (MFIP and MBIP), allowing covalent attachment to the surface. To block unreacted sites and minimize nonspecific adsorption, 100 μL of 1 M ethanolamine (EA) was applied, followed by another PBS rinse. The functionalized sensors were then ready for loop-mediated isothermal amplification (LAMP) reactions and subsequent detection of amplification products.

**Figure 1:**
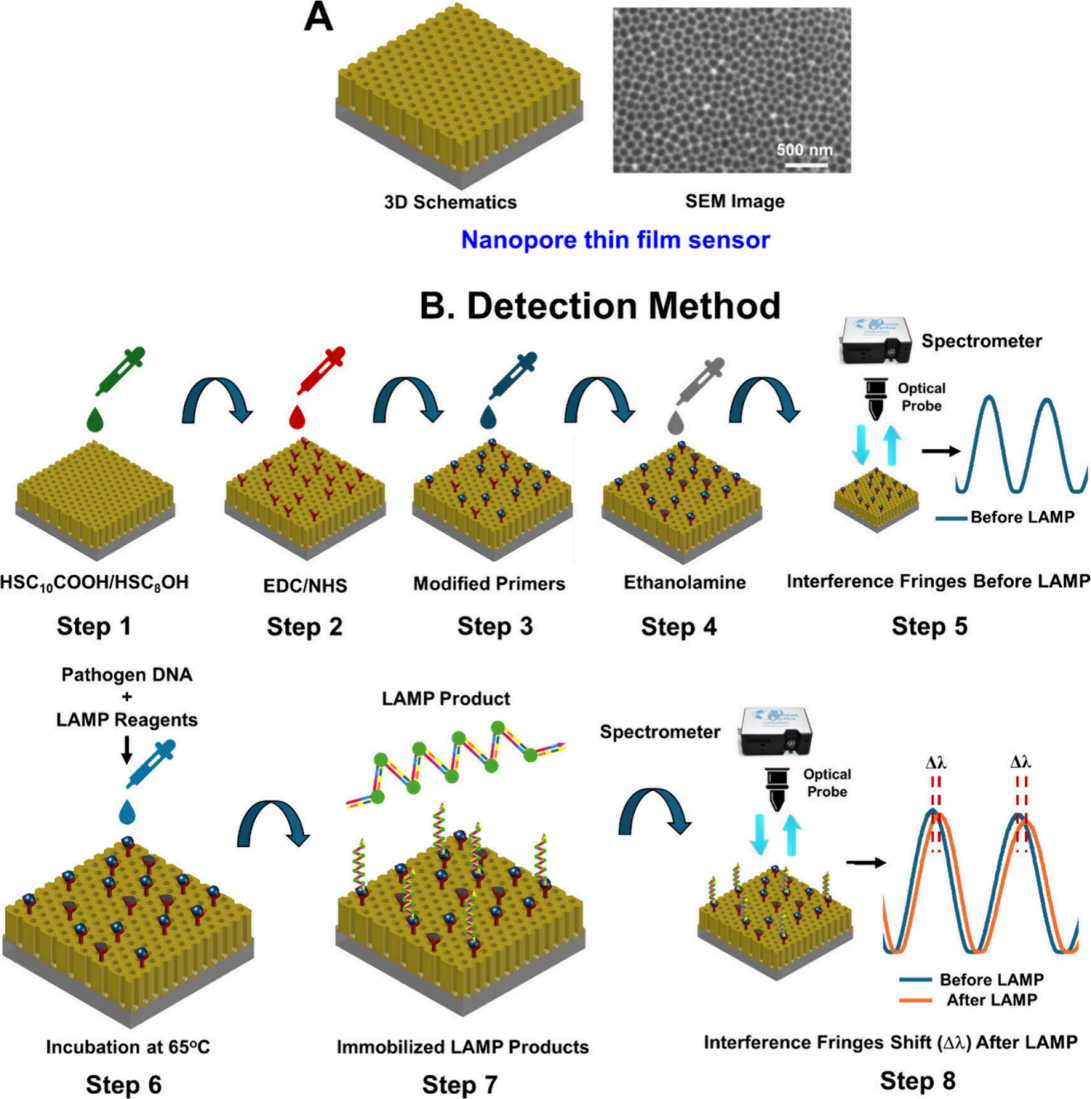
Nanopore thin film sensor chips and LAMP product detection. (A) Schematic of nanopore thin film sensor chips with a SEM image of the nanopores on the sensor surface. (B) Surface functionalization process and the detection mechanism of the LAMP product bound to the nanopore thin film sensor chip.

### 2.5 DNA Extraction

The DNA extraction from *P. infestans* was as described using fresh mycelia.^35^ In brief, the mycelia were collected and ground in liquid nitrogen and homogenized in an extraction buffer (0.2 M Tris-HCl, 0.25 M NaCl, 25 mM EDTA, 0.5% SDS, pH 8.0). After centrifugation, the RNA was removed with 20 μg/mL RNase A by incubation at 65 °C for 30 min. After purification using phenol/chloroform/isoamyl alcohol, the DNA was precipitated with sodium acetate and isopropanol. The DNA quantity and quality were analyzed using a NanoDrop OneC Microvolume UV–vis Spectrophotometer (Thermo Fisher, USA).

### 2.6 Primer Design and Modification

The design of the LAMP primers for detecting the internal transcribed spacer (*ITS*) gene of *P. infestans* was performed using PrimerExplorer V5 (http://primerexplorer.jp/lampv5e/index.html) as previously described.^35^ The unmodified primers, including F3, B3, LoopF, LoopB, FIP, and BIP, were synthesized by Eurofins Genomics LLC (USA), and the 5′-amino-C12-modified FIP and BIP primers were synthesized by Integrated DNA Technologies (USA). Real-time PCR primers were designed using the Primer3Plus qPCR module. The complete primer sequences for the *ITS* gene are listed in Table S1 (in the Supporting Information).

### 2.7 Setup of LAMP Assays

For the standard LAMP reaction, the reaction system includes: 1× Isothermal Amplification Buffer (contains 2 mM MgSO_4_), 6 mM MgSO_4_ (8 mM total), 4.0 mM dNTP mix (1.0 mM Each), 1.6 mM dUTP, 1.6 μmol/L each of FIP and BIP primers, 0.2 μmol/L each of F3 and B3 primers, 0.4 μmol/L each of LoopF and LoopB primers, 1× HNB, 5 U/mL UDG (NEB, USA), 320 U/mL of NEB Bst 2.0 WarmStart DNA Polymerase (NEB, USA), and 1.0 μL of DNA sample at the indicated concentration. The reaction volume was adjusted to 25 μL using nuclease-free ddH_2_O. The LAMP reactions were performed at 65 °C for 40 min or as specified. After LAMP reactions, the amplified LAMP products were analyzed using electrophoresis on a 1.2% agarose gel, and the ethidium bromide-stained gels were visualized and photographed under UV light using the Azure 200 Imaging System (Azure Biosystems, USA).

For the optimization of LAMP reactions conducted on the LAMP-chip, three **approaches** were designed to test the immobilization ratios. **Approach 1**: All of the FIP primers (10 μM) were modified and immobilized on the sensor surface, while the unmodified BIP (40 μM) and the other primers were added directly to the reaction mixture. **Approach 2**: A portion of the modified FIP and BIP primers (10 μM each) were immobilized on the sensor surface, and an equal portion of unmodified FIP and BIP primers (40 μM each) was added to the reaction mixture without immobilization, along with all other primers. **Approach 3**: All of the FIP and BIP primers (10 μM each) were modified and immobilized on the sensor surface, and the remaining primers were added to the reaction mixture. All LAMP reactions on the AAO Sensor chip were performed at 65 °C for 30 min or as specified. After amplification, the LAMP reaction products were analyzed directly using optical detection on the LAMP chips.

### 2.8 Optical Detection of LAMP Products

For the optical measurements, a fiber-optic probe (Ocean Optics, Inc.) was used to deliver white light perpendicularly to the sensor surface. The reflected light was collected by the same probe and directed to a miniature spectrometer (Ocean Optics, Inc.) for analysis (**Step 5** in Figure 1B). Initially, a reference optical signal was recorded using a fresh, nonfunctionalized sensor. Subsequently, optical signals were collected after each step of surface chemical functionalization, continuing through to the immobilization of the inner primers on the sensor surface (**Step 8** in Figure 1B). Because primers are short DNA oligonucleotides, they are too small to be visualized clearly by standard SEM. Detecting surface-bound DNA typically requires advanced imaging methods such as high-resolution FE-SEM or TEM often with heavy-metal labeling or other contrast-enhancement techniques.^38,39^ Optical interference spectroscopy offers a straightforward and sensitive method to verify surface immobilization, which is given in Figure S1 (in the Supporting Information). Following functionalization, the sensor chamber was filled with the LAMP reaction mixture and incubated at 65 °C for 30 min or as specified. Upon completion of the reaction, the chamber was thoroughly rinsed multiple times before final optical measurements were taken.

### 2.9 Real-Time PCR Assays

Real-time PCR was performed using a dilution series of *P. infestans* US-23 genomic DNA as templates. Each 10 μL reaction mixture contained 0.1 μL of each forward and reverse primer (40 μM), 5 μL of KAPA SYBR FAST qPCR Master Mix (ROX Low, Roche, USA), 0.1 μL of DNA template at the specified concentration, and nuclease-free water to adjust the total volume to 10 μL. Reactions were prepared on ice. PCR amplification was carried out on a QuantStudio 6 Flex Real-Time PCR System (Thermo Fisher, USA) with the following cycling conditions: initial denaturation at 95 °C for 3 min; 40 cycles of 95 °C for 3 s and 65 °C for 30 s. Immediately following amplification, melting curve analysis was performed with these steps: 95 °C for 15 s, 65 °C for 1 min, a gradual temperature increase to 95 °C at 0.05 °C/s, and a final hold at 95 °C for 15 s. Each real-time PCR assay was conducted in triplicate. A calibration curve was generated from the series of DNA dilutions with known concentrations, and quantification cycle (Cq) values were plotted using Python 3. For real-time PCR validation of DNA amounts in the pseudo-carryover experiments and LAMP-chip assays, bar plots were generated using the 2^-△△Cq^ method.

### 2.10 Data Collection and Analysis

Optical signals were collected from the sensor both before and after the LAMP reaction. The peak shifts in the interference fringes of these signals were averaged to determine the transducing signal (i.e., the optical signal shift) generated by the sensor. Control experiments revealed minor spectral shifts, likely resulting from nonspecific interactions such as the binding of reaction components to the sensor surface, occasional extension of immobilized primers, or nonspecific hybridization between immobilized primers and DNA templates or free primers. These shifts were treated as background noise and subtracted from the measured results. Data are presented as mean ± standard deviation (SD), and all experiments were independently repeated at least three times.

### 2.11 Real-Time LAMP Assays

The real-time LAMP assays were conducted as described using a reaction volume of 10 μL.^35^ The reactions were mixed on ice and contained 1× Isothermal Amplification Buffer (NEB, USA), 10 mM MgSO_4_ 2.8 mM dNTP Mix (0.7 mM Each), 2.8 mM dUTP, 3.2 μM FIP and BIP (1.6 μM Each), 0.4 μM F3 and B3 (0.2 μM Each), 0.8 μM LoopF and LoopB (0.4 μM Each), 1× EvaGreen (Biotium, USA), 0.5 μM ROX, 1× HNB, 0.05 U UDG (NEB, USA), 3.2 U *Bst* 2.0 WarmStart DNA polymerase (NEB, USA), 0.8 M betaine, and 1 μL of the DNA sample at the indicated concentrations, adjusted to 10 μL volume with ddH_2_O. The real-time LAMP was conducted on a QuantStudio 6 Flex Real-Time PCR System (Thermo Fisher, USA) using the following conditions: 25 °C for 2 min, followed by 260 cycles of 65 °C for 30 s, plus a melting curve analysis: 95 °C for 15 s, 60 °C for 1 min, a gradual increase to 95 °C (0.05 °C/s), and 95 °C for 15 s, followed by a polymerase inactivation step at 85 °C for 20 min. Real-time LAMP reactions with ddH_2_O as template were always included as negative controls. All real-time LAMP assays were performed in 3–6 replicates. A calibration plot was generated from quantification time (Tq) values using a series of concentrations of *P. infestans* DNA. The Tq values were plotted using a Python3 script.

## 3 Results and Discussion

### 3.1 Determining the Optimal Primer Immobilization Strategy

To optimize the transducing signal, we evaluated three different approaches by adjusting the ratios and concentrations of both immobilized and unmodified FIP and BIP primers, noting that primers were modified only when immobilized on the sensor surface, while all primers in the solution were unmodified. These two primers were identified as having the most significant impact on LAMP detection.^35^ As aforementioned, in **Approach 1**, all FIP primers were immobilized on the sensor surface, with all BIP primers and other primers remaining unmodified in the solution. In **Approach 2**, a portion of both modified FIP and BIP primers were immobilized on the sensor, while the remaining FIP and BIP primers, as well as all other primers, remained unmodified in the solution. In **Approach 3**, all FIP and BIP primers were immobilized, with all other primers unmodified in the solution.

Each approach was tested using two concentrations of *P. infestans* DNA: 10 fg/μL and 0 fg/μL (control). Unless otherwise specified, the concentration of immobilized primers was 10 μM for both modified FIP and BIP when used. In **Approach 1**, instead of adding 1 μL each of 40 μM FIP and BIP, the LAMP solution contained 2 μL of 40 μM unmodified BIP only, while all other LAMP reagents were kept constant across approaches. Transducing signals were measured from four sensors per approach, with the results presented in Figure 2.

**Figure 2:**
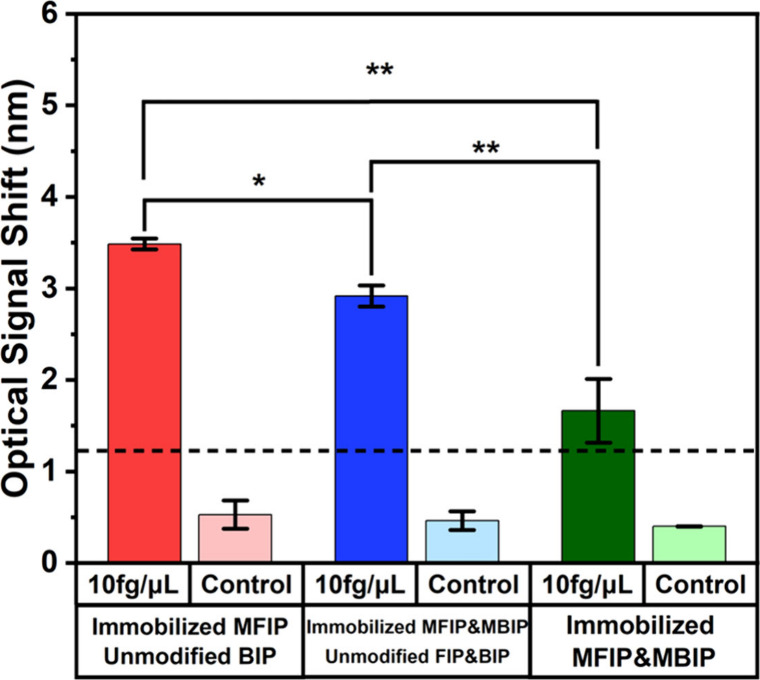
Optical signals of LAMP chip assays under different primer immobilization strategies. Three approaches were performed: (1) Approach 1: All FIP primers immobilized on the sensor surface, while BIP and other primers remained unmodified in solution; (2) Approach 2: A portion of FIP and BIP primers immobilized on the sensor, with the remaining FIP, BIP, and other primers unmodified in solution; and (3) Approach 3: All FIP and BIP primers immobilized on the sensor, with all other primers unmodified in solution. Assays were performed using 10 fg/μL *P. infestans* US-23 DNA template and a negative control lacking *P. infestans* DNA. Three independent samples were measured for each condition (*n* = 3). Error bars represent the standard deviations. * indicates *p* = 0.002, and ** indicates *p* < 0.001.

In the absence of *P. infestans* DNA (0 fg/μL control), all three approaches produced comparable transducing signals, with an average optical interference fringe shift of approximately ∼ 0.5 nm. However, in the presence of 10 fg/μL *P. infestans* DNA, the transducing signals varied significantly among the approaches. **Approach 1** generated the highest signal at approximately **3.48 nm**, followed by **Approach 2** at **2.93 nm**, and **Approach 3** at **1.66 nm**, respectively.

The dotted line in Figure 2 represents the cutoff transducing signal, which indicates the minimum signal required to reliably distinguish a sample from the control. Signals below this threshold were considered indistinguishable from background noise and therefore insufficient for reliable detection, while signals above it are considered detectable. The cutoff value was calculated using the **3σ method**, defined as the highest average signal from the control (∼0.78 nm) plus three times the corresponding standard deviation (∼0.13 nm), resulting in a cutoff of approximately **1.2 nm**. One way ANOVA complemented by posthoc comparison determined a statistically significant difference (*p* ≤ 0.002) among the readings in the mentioned three approaches. Based on this criterion, **Approach 1** produced the highest and most distinguishable signal, making it the **preferred approach** for detecting pathogen DNA.

### 3.2 Determining the Optimal Concentration of Immobilized Primers

We investigated the effect of varying concentrations of 5′-amino-C12-modified primers immobilized on the sensor surface to optimize the transducing signal. Using 10 fg/μL *P. infestans* DNA in the LAMP solution, we tested three immobilized FIP concentrations, 40 μM, 20 μM, and 40 μM, under **Approach 1**, while keeping the unmodified BIP primer concentration in the LAMP solution constant at 10 μM. The results showed that immobilizing **20 μM** FIP produced the highest transducing signal (∼**4.33 nm**), whereas **40 μM** resulted in a lower signal (∼**2.76 nm**), as shown in Figure 3A.

**Figure 3:**
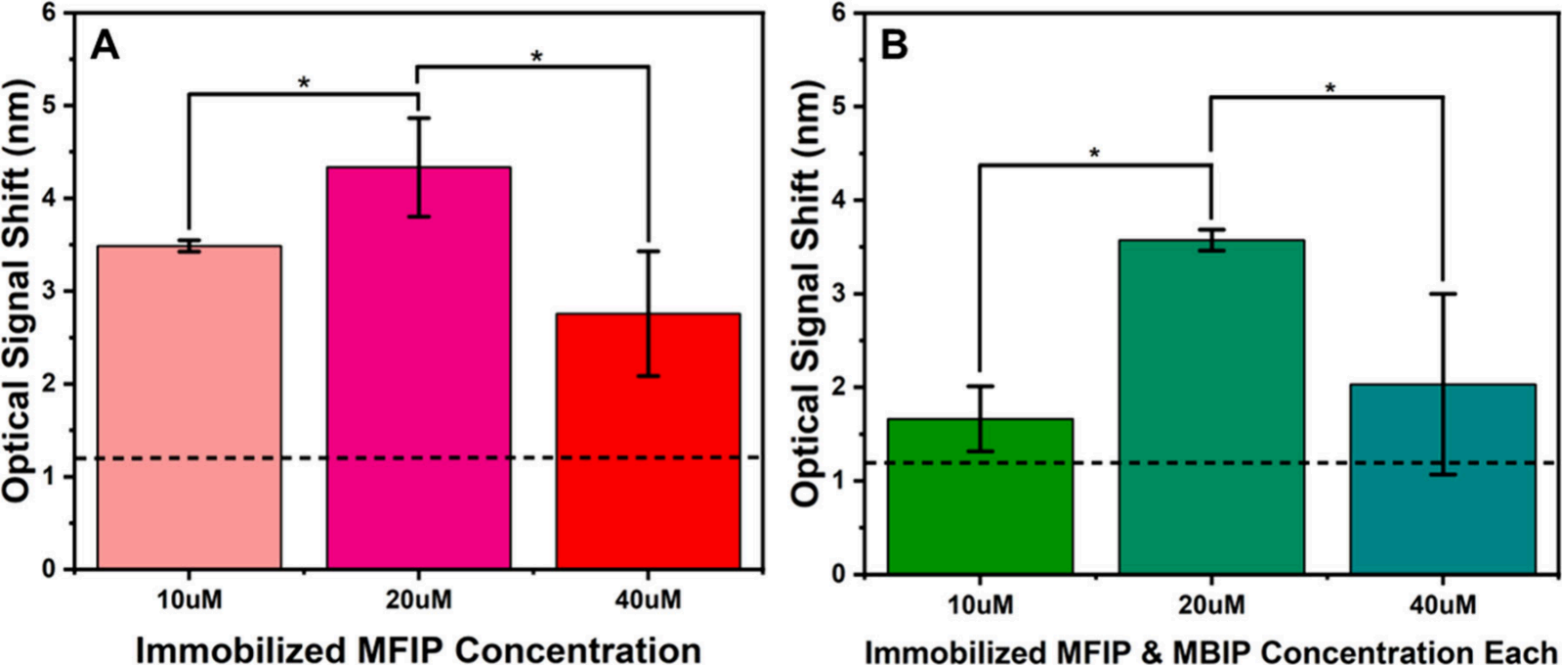
Optical signals from LAMP chip assays using different concentrations of immobilized inner primers. (A) Signals with 10 μM, 20 μM, and 40 μM immobilized FIP. In all cases, 40 μM unmodified BIP was added to the LAMP solution. (B) Signals with 10 μM, 20 μM, and 40 μM of each modified FIP and BIP (MFIP and MBIP) were immobilized with no FIP or BIP in the LAMP solution. LAMP chip assays were performed using 10fg/μL *P. infestans* US-23 DNA template. Each condition was tested in triplicate (*n* = 3). Error bars represent the standard deviations, and * indicates *p* < 0.05.

A similar trend was observed in **Approach 3**, where all FIP and BIP primers were immobilized on the sensor surface. The highest transducing signal (∼**3.57 nm**) was achieved when both primers were immobilized at **20 μM**. Increasing the concentration to **40 μM** led to a reduced signal (∼**2.03 nm**), as shown in Figure 3B. In all experiments, the LAMP reaction time was kept constant at **30 min**. One way ANOVA with subsequent posthoc analysis concluded that the observed variations in the optical signal shift were significantly associated with the changes in immobilized primers concentrations (*p* < 0.05).

As aforementioned, in both **Approach 1** and **Approach 3**, the relationship between immobilized primer concentration and the optical transducing signal followed a similar pattern. As the immobilized primer concentration increased from 10 μM to 20 μM, the average optical shift also increased. This trend is intuitive, as higher primer densities provide more binding sites for the LAMP amplicons, leading to larger interference fringe shifts. However, further increasing the primer concentration to 40 μM caused a reduction in the optical signal (Figure 3). Because LAMP generates large, branched, high-molecular-weight DNA structures, excessive primer density (40 μM) on the surface likely introduces steric hindrance that restricts the accessibility of functional binding sites. Several previous studies have confirmed this steric blocking effect for surfaces densely coated with antibodies,^40^ aptamers,^41^ and oligodendrocytes.^42^ Taken together, these results indicate that 20 μM of FIP and BIP is the optimal immobilization concentration for both approaches. Additionally, immobilizing only FIP reduced surface crowding of the LAMP products and produced the highest optical transducing signal.

### 3.3 Assessing Reduced Reaction Time for LAMP Chip Detection

Using 20 μM of immobilized inner primers on the sensor surface, we investigated the optimal LAMP reaction time for detecting plant pathogens. The reactions were carried out at 65 °C, with reaction times ranging from 10 to 30 min at 5 min intervals. For comparison, one set of sensors was prepared with 10 μM of immobilized inner primers, while another set used 20 μM of modified inner primers. In all cases, the LAMP solution contained a constant concentration of 40 μM unmodified BIP, and these solutions were applied to sensors with only FIP immobilized.

As shown in Figure 4A, sensors with 20 μM immobilized FIP produced a transducing signal of approximately 3 nm within 20 min, well above the reliable detection threshold. In contrast, the set of sensors with both 20 μM FIP and BIP immobilized reached a similar signal level after 25 min (Figure 4B). A DNA template concentration of 10 fg/μL from *P. infestans* was used in all LAMP reactions. These results indicate that the reaction time for sample detection can be optimized to as little as 20 min, a significant reduction compared to the 30 min or even 60 min typically required for conventional LAMP assays.^43–45^

**Figure 4:**
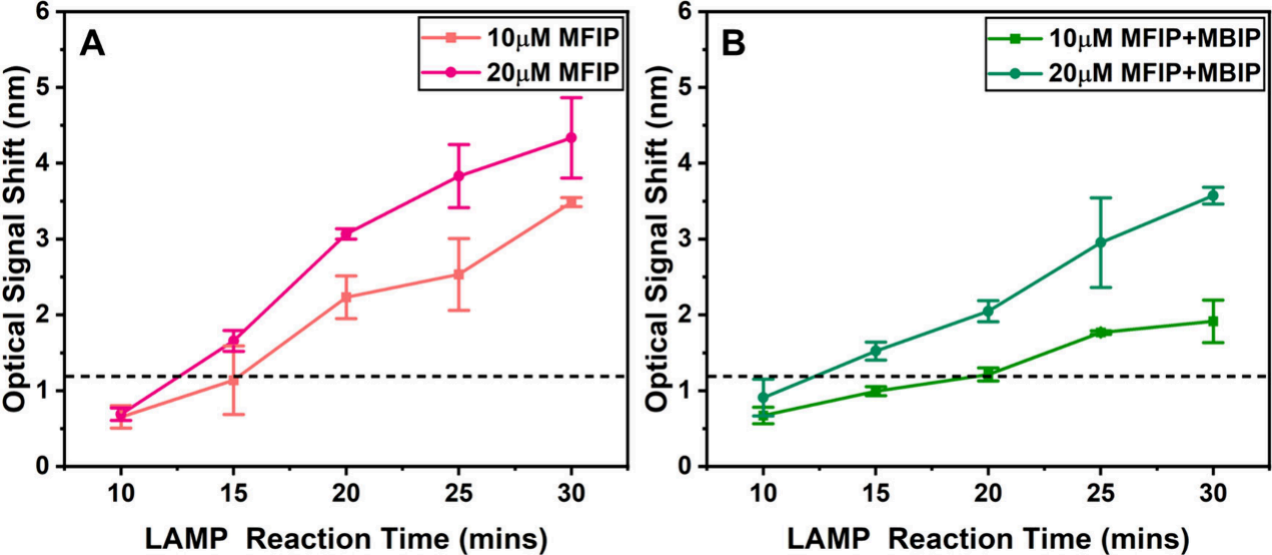
Optical signals over LAMP reaction time. (A) All modified FIP (MFIP) primers were immobilized on the sensors with 40 μM unmodified BIP added to the LAMP solution. (B) All modified FIP and BIP (MFIP and MBIP) primers were immobilized on the surface of the sensors with no FIP or BIP included in the LAMP solution. The LAMP mixture contained 10 fg/μL of *P. infestans* US-23 DNA template.

### 3.4 Evaluating the Detection Limit of the LAMP Chip

To evaluate the sensitivity of detection within a shortened reaction time of 20 min, a series of *P. infestans* DNA template concentrations were tested in the LAMP solution. In these experiments, each sensor surface was functionalized with 20 μM immobilized FIP, while the LAMP solution contained 40 μM BIP. The DNA template concentration started at 1 ng/μL and was serially diluted 10-fold down to 0.1 fg/μL. A control sample containing no DNA template was also included to assess background signal levels.

As shown in Figure 5, a clear transducing signal of approximately 3 nm was observed with DNA concentrations as low as 1 fg/μL. At concentrations below this threshold, the signal dropped to below 1 nm, comparable to the control, indicating that the limit of detection is 1 fg/ μL. These results demonstrate that the system can reliably detect target DNA at femtogram levels within just 20 min of reaction time.

**Figure 5:**
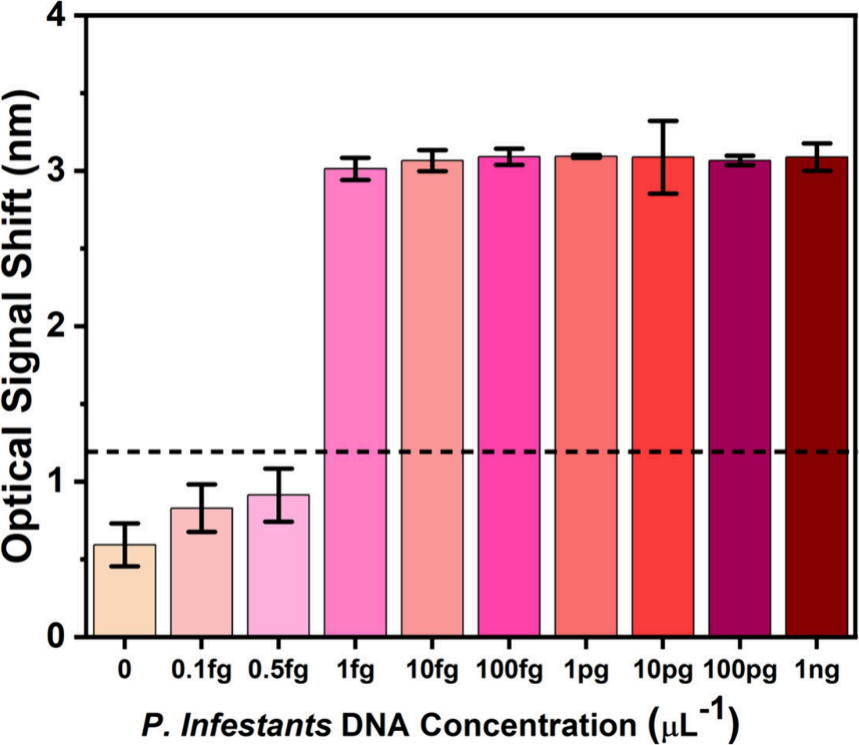
Optical signals from LAMP-chip assays across *P. infestans* DNA concentrations. Each LAMP chip assay was performed using serial dilutions of *P. infestans* US-23 DNA for 20 min. The immobilized MFIP concentration was set to 20 μM, and 40 μM BIP was added in the LAMP solution. All other primers and components remained constant.

For comparison, real-time PCR was used to detect *P. infestans* DNA. Based on the measurements in Figure S2A,B (in the Supporting Information), ANOVA and posthoc analyses indicate that real-time PCR has a detection limit in the range of 40–100 fg/μL. In comparison, real-time LAMP assays showed an effective detection limit of 10 pg/μL (Figure S2C). Additionally, Table S2 (in the Supporting Information) presents a comparison of recent *P. infestans* detection techniques, including their reaction times and limits of detection (LOD). Notably, our LAMP-chip demonstrates improved performance in both reaction time and LOD.

### 3.5 Evaluating Carryover Contamination of the LAMP Chip

To evaluate potential carryover contamination and confirm surface-bound DNA capture on the LAMP chip, two distinct assays were conducted: a pseudocarryover assay and a surface-bound DNA assay. Each assay involved a two-step process. The workflow for preparation and tests of two carryover contamination assays is illustrated in Figure S3 in the Supporting Information.

In the first step of the pseudocarryover assay, the standard EDC-NHS surface functionalization was deliberately omitted. As a result, no modified FIP or BIP primers were immobilized on the sensor surface. Instead, two different LAMP reaction setups were used. The first setup contained 4 μL of 20 μM modified FIP and 2 μL of 40 μM unmodified BIP per 25 μL of LAMP solution. The second setup included 2.5 μL of 20 μM modified FIP and 2.5 μL of 20 μM modified BIP per 25 μL of LAMP solution. Both formulations also contained 0.5 μL of 10 fg/μL DNA from *P. infestans*. Sensor chips were immersed in these LAMP solutions and incubated at 65 °C for 30 min. Since no surface chemistry was employed, the DNA samples were not expected to bind to the nanopores of the sensor. Consequently, there should be minimal change in the optical interference fringes before and after incubation. As shown in Figure 6A, the resulting signals remained below the detection threshold, confirming that DNA remained in the LAMP solution and did not adhere to the sensor surface. This free-floating DNA represents a potential source of carryover contamination. In contrast, when the EDC-NHS surface activation was applied, followed by immobilization of modified FIP alone or in combination with modified BIP as described in Section 2.8, significant interference fringe shifts were observed (also shown in Figure 6A), confirming successful DNA binding to the sensor surface.

**Figure 6:**
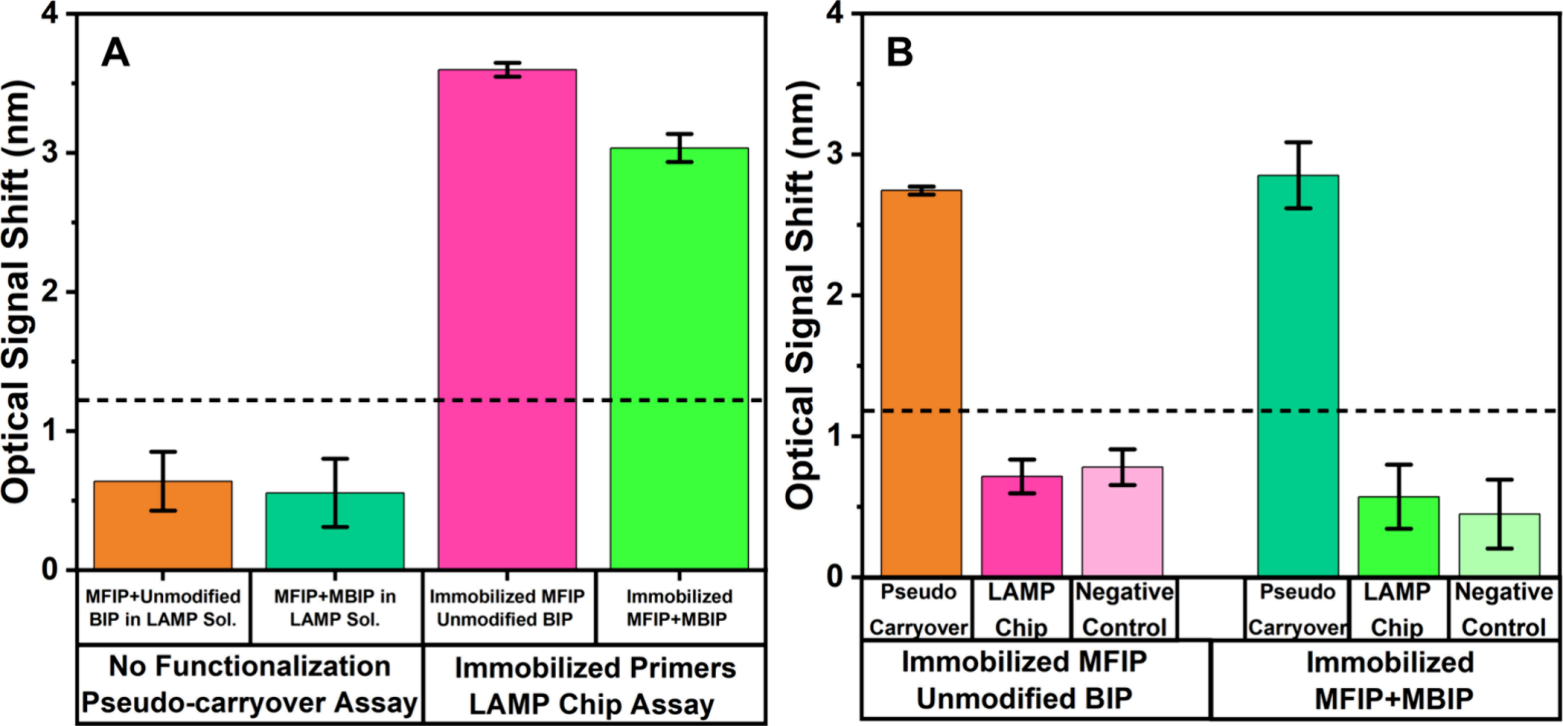
Optical signals from pseudocarryover and surface-bound DNA tests in LAMP-chip assays. (A) Optical signals from the first step of the pseudocarryover assay and LAMP Chip (surface-bound DNA) assay. For the pseudo carryover assay, LAMP was performed on the sensor with no functionalization of the sensor surface. Consequently, all amplified DNA remained free in the supernatant and did not attach to the sensor, and the optical signals before and after incubation were below the cutoff value after stand washing. In contrast, for the LAMP chip (surface bound DNA) assay, LAMP chip had immobilized primers on the surface. The amplified LAMP products were attached to the sensor, resulting in high optical signals. The supernatant contained little or no DNA. (B) Optical signals using the supernatant of the first tests described in (A). LAMP solutions contained 1 μL of the supernatants from the pseudocarryover assay and LAMP chip assay. The supernatant from the pseudocarryover resulted in high optical signals. In contrast, the surface-bound supernatant from LAMP chip generated background-level optical signals, which are comparable to those from negative control.

In the second step, we applied the standard EDC-NHS surface chemistry to immobilize FIP and/or BIP primers onto the sensor surface, as previously described. Instead of using fresh DNA from *P. infestans*, we introduced a small volume (∼1 μL) of LAMP product obtained from the first step. This included both the pseudocarryover and the surface-bound LAMP chip supernatant. Because the supernatant from the pseudocarryover assay is expected to contain DNA residue, unlike the supernatant from the LAMP chip, we anticipated a noticeable optical transducing signal in the sensors to which the pseudocarryover product was applied. As shown in Figure 6B, this expectation was confirmed: a significant transducing signal was observed in sensors to which the pseudocarryover supernatant was applied, indicating the presence of amplifiable free DNA template. We anticipated that the supernatant from the surface-bound LAMP chip assay would contain little to no free DNA and would not produce a significant transducing signal in the highly sensitive LAMP chip assay. As expected, sensors to which the LAMP chip supernatant was applied showed minimal signal, similar to the negative control lacking *P. infestans* DNA template, indicating that the residual free DNA, the main cause of carryover contamination, was minimal or absent. These results demonstrate that the sensor surface functionalized with EDC-NHS and modified FIP and BIP primers effectively captures DNA on the chip and significantly reduces, if it does not entirely eliminate, the risk of carryover contamination.

To further validate the extent of residual free DNA in the pseudocarryover experiment and LAMP-chip assays, we performed real-time PCR analysis targeting LAMP amplicons using the supernatants collected from the pseudocarryover experiment and from two LAMP-chip approaches (**Approach 1** and **Approach 3**). As shown in Supplemental Figure S4, the **Approach 1** LAMP-chip reduced the amount of carryover DNA in the supernatant to approximately 12% of that observed in the pseudocarryover condition. In contrast, the **Approach 3** LAMP-chip effectively removed detectable carryover DNA, indicating that its immobilization chemistry and surface-binding efficiency prevented the release of amplification products into the solution. These results provide quantitative confirmation that the optimized LAMP-chip approaches substantially suppress free amplicon carryover, strengthening the biosafety and contamination-resistance advantages of the solid-phase LAMP chip format.

All above results highlight a key advantage of the LAMP-chip system: its inherent ability to minimize carryover contamination by physically capturing amplified DNA on the sensor surface. In conventional LAMP workflows, aerosolized or residual amplicons can easily contaminate subsequent reactions, leading to false positives and reduced assay reliability. Our findings demonstrate that the functionalized sensor surface significantly reduces free DNA in the reaction supernatant, thereby limiting the primary source of carryover contamination. Furthermore, the comparison between pseudocarryover and surface-bound assays confirms that the immobilization chemistry effectively traps amplification products within the nanoporous structure, preventing their release into the surrounding solution. This surface-mediated containment not only improves diagnostic robustness but also provides a distinct biosafety advantage over conventional LAMP assays or standard LAMP-based sensor platforms, which remain highly vulnerable to contamination during handling, pipetting, or postamplification processing.

### 3.6 Optimized LAMP Chip Assay for Plant Pathogen Detection

To evaluate the point-of-care potential of the sensor, the optimized LAMP chip assay was tested using tissue lysates from *P. infestans*–infected and healthy potato plants. Samples were collected from plants exhibiting various infection symptoms from mild to moderate along with matched healthy controls. Lysates were anonymized and labeled as groups **1–8 (noninfected)** and **A–H (infected)**. Figure 7 shows the transducing signals from noninfected (1–8) and infected (A-H) groups, with purified DNA results included for comparison. Using the optimized assay (20 μM immobilized modified FIP on the chip surface and 40 μM BIP in the LAMP solution), infected samples (A-H) produced an average transducing signal of ∼ 3.10 nm, comparable to that of purified *P. infestans* DNA at ≥ 1 fg/μL. In contrast, noninfected samples (1–8) generated transducing signal of <1 nm, similar to the no-template control and ≤ 1 fg/μL DNA samples. Each lysate was analyzed in triplicate, and the LAMP reaction concluded within 20 min. A standard 40 min LAMP reaction also detected *P. infestans* DNA in groups **A-H** as shown in Figure S5 (in the Supporting Information). Collectively, these results demonstrate that the optimized LAMP chip assay retains high specificity and sensitivity even in crude plant extracts, underscoring its promise as a simple, rapid, and effective point-of-care platform for early plant-pathogen detection. Figure S6 (in the Supporting Information) presents the field-ready workflow, along with operational guidelines, for conducting plant-pathogen detection with the LAMP-on-chip system. All components fit within a small carry-case for point-of-care deployment.

**Figure 7:**
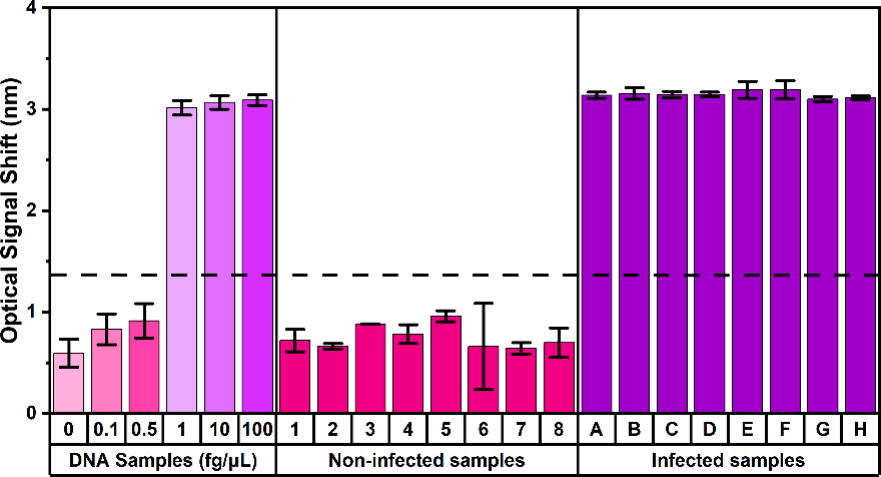
Detection of pathogen from late-blight infected potato plants with the optimized LAMP-chip assay. For noninfected samples (*groups 1–8*), the transducing signals are <1 nm, comparable to the transducing signals of the negative control (0 fg/μL) and those of <1 fg/μL purified DNA samples. For infected samples (*groups A–H)*, the transducing signals are >3 nm, closely matching the transducing signals of samples with >1 fg/μL purified DNA. The dashed line indicates the cutoff value at 1.2 nm defined by the 3σ method. Error bars were derived from standard deviation calculation from 3 readings for each sample.

## 4 Conclusions

In this study, we systematically investigated how primer concentration and the ratio of immobilized primers influence the transducing signals generated by the LAMP chip and further assessed the platform’s capability to reduce or eliminate carryover contamination. Our goal was to maximize these parameters to achieve the highest possible sensitivity and reliability in detecting target pathogen DNA. Through extensive testing, we determined that the most effective configuration involved immobilizing the FIP primer on the sensor surface while using the unmodified BIP primer in the LAMP reaction solution. This setup enabled the detection of *P. infestans* DNA at a low concentration of 1 fg/μL, producing transducing signals up to 3.48 nm after a 30 min LAMP reaction. Further optimization revealed that immobilizing both modified FIP and modified BIP primers at a concentration of 20 μM yielded the best overall performance. Beyond primer optimization, we also evaluated the minimal reaction time required to reliably detect the pathogen using our LAMP-chip system. The optimized approach allowed for the detection of *P. infestans* in under 20 min, approximately a 33% reduction compared to the standard 30 min LAMP assays, demonstrating not only enhanced sensitivity but also significantly faster turnaround times. A key advantage of our approach is the functionalization of the sensor surface with EDC-NHS chemistry to covalently immobilize the modified primers. This immobilization effectively captures amplified DNA directly on the chip, thereby substantially reducing or even eliminating the risk of carryover contamination that often plagues conventional nucleic acid amplification methods.

Given its low cost, straightforward operation, and high sensitivity and specificity, this LAMP-chip platform holds great promise for point-of-care testing (POCT) applications. The rapid and label-free optical detection allows for immediate and accurate identification of plant pathogens onsite, enabling timely intervention and disease management. Moreover, the significant reduction in carryover contamination enhances the reliability of repeated assays, making it a valuable tool for monitoring pathogen prevalence and evaluating the effectiveness of therapeutic treatments in agricultural settings. This technology has the potential to transform plant disease diagnostics by providing farmers, agronomists, and researchers with a portable, easy-to-use device that delivers high-precision results rapidly, ultimately helping safeguard crop health and improve agricultural productivity worldwide.

## Supplementary Material



## Notes

The LAMP and PCR primer sequences for the *P. infestans ITS* gene; monitoring of the surface functionalization and LAMP reaction on the nanopore thin-film biosensor; real-time PCR and LAMP assay test of *P. infestans*; comparison of detection techniques, reaction times, and limits of detection (LOD) for *P. infestans* reported in recent studies; schematic workflow of the carryover contamination test; real-time PCR of the pseudocarryover and LAMP chip supernatants; LAMP tests for potato infected with *P. Infestans* US-23; and field-ready workflow for rapid plant-pathogen detection using a LAMP-on-chip system (PDF)
