# Supplementary material for: A Systematically Enhanced LAMP Chip for Rapid, Sensitive, and Contamination-Free Pathogen Detection
Source: ACS Meas Sci Au. 2025 Dec 25;6(1):179–88. doi: 10.1021/acsmeasuresciau.5c00155 (PMC12921607; doi:10.1021/acsmeasuresciau.5c00155)
Supplement: Supplementary file 1 [file tg5c00155_si_001.pdf]

Supporting Information

A Systematically Enhanced LAMP Chip for Rapid, Sensitive, and Contamination-Free Pathogen Detection

Sajid Uchayash[1,+], Jinping Zhao[2,+], Md Iqbal Kabir[1], Junqi Song[2,3*], Long Que[1,*]

[1]Department of Electrical and Computer Engineering,

Iowa State University, Ames IA 50011, USA

[2]Texas A&M AgriLife Research Center at Dallas

Texas A&M University System, Dallas, TX 75252, USA

[3]Department of Plant Pathology & Microbiology

Texas A&M University, College Station, USA, 75252

[+]Equal contribution

*Correspondence to L. Que (Email: lque@iastate.edu) and J. Song (Email: junqi.song@ag.tamu.edu)

Table S1. The LAMP and Real-time PCR primer sequences for the *P. infestans ITS* gene

**Primer Number**	**Sequence**	**Length (nt)**	**Annotation**
Lamp47	CAAAGGACTCGCAGTCGGTCCGG AGGAGATGCCAGATGTGA	39	FIP for *P. infestans ITS* gene
Lamp48	AAGTGGTGGCATTGCTGGTTGTTT TCTCCATTAACGCCGCAG	40	BIP for *P. infestans ITS* gene
Lamp49	GGAAACCAACCGCAAGACACT	20	LoopF for *P. infestans ITS* gene
Lamp50	GACGCTGCTATTGTAGCGAGTTG	20	LoopB for *P. infestans ITS* gene
Lamp51	TTCTCCCTTCCGTGTAGTCG	20	F3 for *P. infestans ITS* gene
Lamp52	GGCAACCATACCACGAATCG	18	B3 for *P. infestans ITS* gene
Lamp53	AMC12- CAAAGGACTCGCAGTCGGTCCGG AGGAGATGCCAGATGTGA	39	FIP for *P. infestans ITS* gene with 5'-Amino C-12
Lamp54	AMC12- AAGTGGTGGCATTGCTGGTTGTTT TCTCCATTAACGCCGCAG	40	BIP for *P. infestans ITS* gene with 5'-Amino C-12
ojp2715	tattgcacttccgggttagtcc	22	Real-time PCR forward primer for *P. infestans ITS* gene
ojp2716	tgttgtacggacactgatacagg	23	Real-time PCR reverse primer for *P. infestans ITS* gene

Primers are short DNA oligonucleotides and are far too small to be visualized by standard SEM. Detecting surface-bound DNA typically requires advanced imaging methods—such as high-resolution FE-SEM or TEM—often with heavy-metal labeling or other contrast-enhancement techniques [14,15]. Optical interference spectroscopy offers a straightforward and sensitive method to verify successful surface immobilization. The complete functionalization workflow is shown in **Figure 1**. As an example, **Figure S1A** displays the interference fringe evolution at each modification step compared with the blank nanopore thin-film surface, and **Figure S1B** quantifies the corresponding shifts. Incubation with HCS10COOH/HSC8OH produced a ~0.3 nm shift, followed by an additional ~1.5 nm shift after EDC/NHS activation. Immobilizing 20 µM FIP primer generated a further ~1 nm displacement, confirming successful attachment of the modified oligonucleotides. Ethanolamine (EA) blocking caused only a ~0.25 nm shift, establishing the "before LAMP" baseline. Upon introducing 10 fg/µL purified target DNA, the LAMP reaction produced a pronounced ~3 nm shift, as expected due to the formation of large, hyperbranched DNA products that significantly increase the optical path length within the nanopore film. In contrast, samples at or below the amplification threshold (≤1 fg/µL) and the no-template control showed only ~0.6 nm shifts, consistent with unsuccessful amplification.

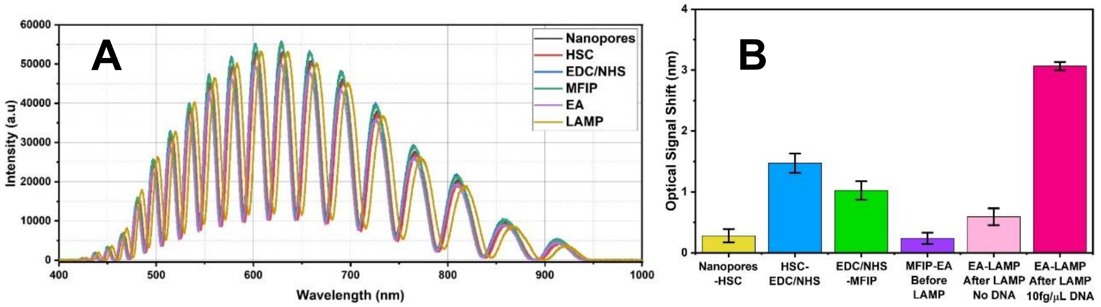

Figure S1. Monitoring of the surface functionalization and LAMP reaction on the nanopore thin-film biosensor. (A) A demonstration of the corresponding interference fringe evolution at each surface functionalization step. "Nanopore" marks the initial interference fringes of the nanopore thin film. "HSC" designates the fringes after $HCS_{10}COOH/HSC_8OH$ incubation. "MFIP" denotes the fringes after primer immobilization. "EA" and "LAMP" refer to fringes before and after the LAMP reaction, respectively. The LAMP reaction was performed with 10fg/µL purified DNA. A conspicuous shift is observed before and after the LAMP reaction. (B) Quantification of the interference fringe shift observed between sequential functionalization steps. The consecutive steps are indicated.

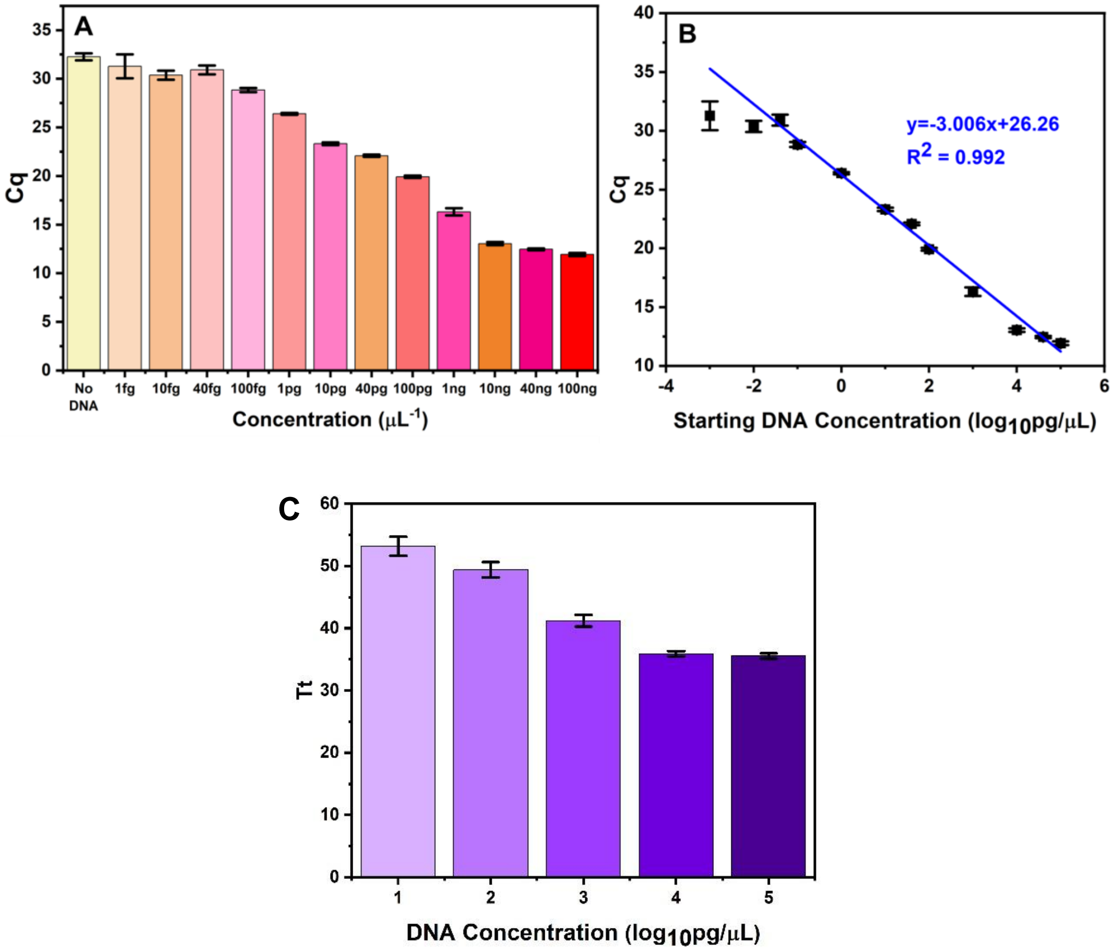

Figure S2. Real-time PCR and real-time LAMP detection of *P. infestans*. (A) Detection of real-time PCR of *P. infestans*. A series of dilutions were performed of purified genomic DNA as template. At least three replicates were performed for each concentration. (B) Real time PCR calibration curve with respect to logarithmic pg/μL concentration variation. (C) Real-time LAMP detection of *P. Infestans*. Each dilution of *P. Infestans* DNA template was tested at least three replicates.

Table S2: Comparison of detection techniques, reaction times, and limits of detection (LOD) for *P. infestans* reported in recent studies [1-5].

Detection Technique	Limit of Detection (LOD)	Reaction time	Reference
Colorimetry	1 pg/µL	70 min	[1]
Conventional PCR	10 pg/µL	40 min	[2]
Real-time LAMP	1 pg/µL		
Droplet digital PCR	100 fg/µL		
SYBR Green LAMP read on the mReader	584 fg/µL		
Colorimetry	2 pg/µL	60 min	[3]
Fluorescence-based	1 pg/µL	~35 min	[4]
Optical LAMP chip sensor	1 fg/µL	30 min	[5]
Optimized Optical LAMP chip sensor	1 fg/µL	20 min	This work

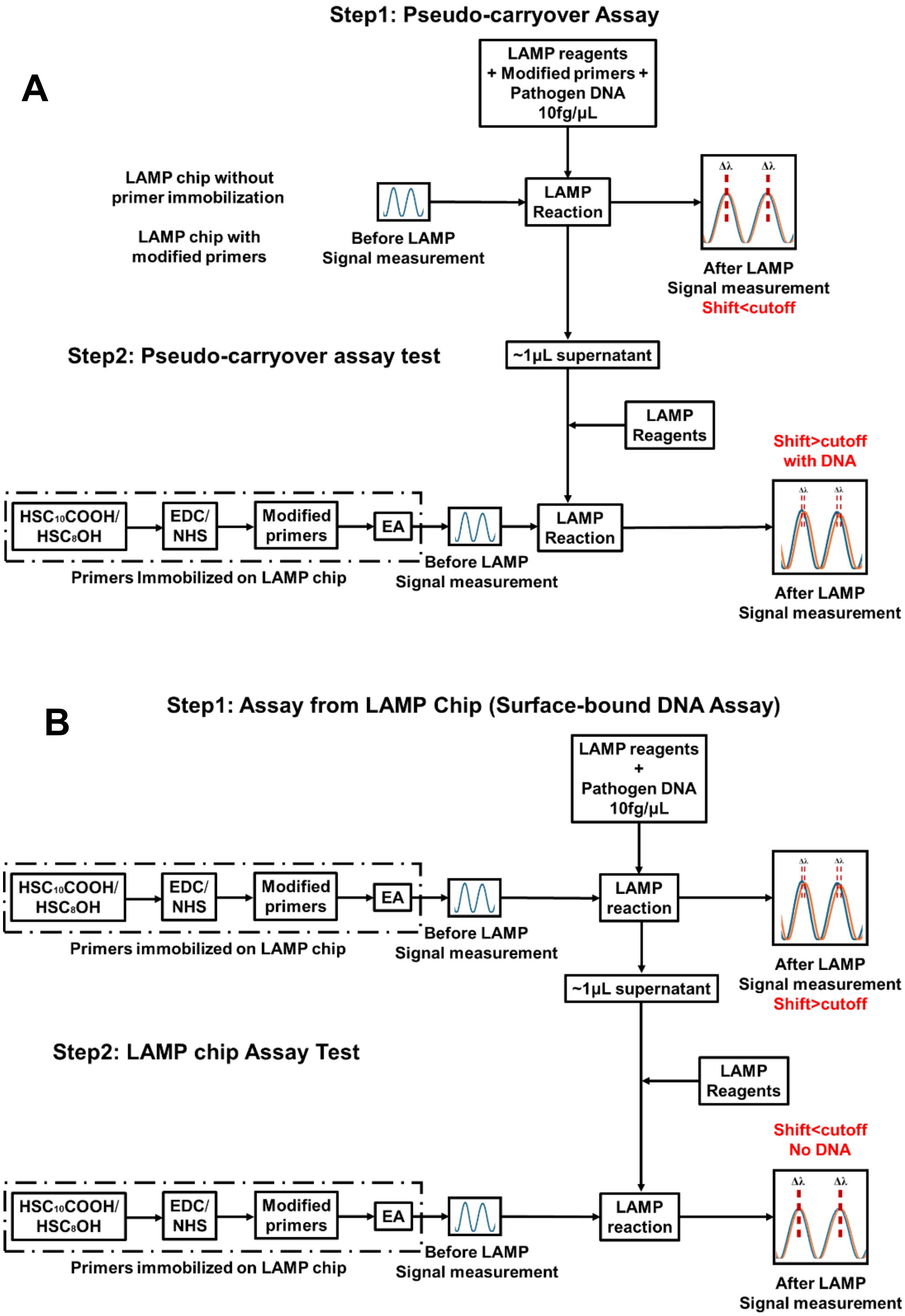

Figure S3: Schematic workflow of the carryover contamination test. (A) Preparation and execution of the pseudo-carryover assay. (B) Preparation and execution of the surface-bound DNA assay (LAMP chip assay).

.

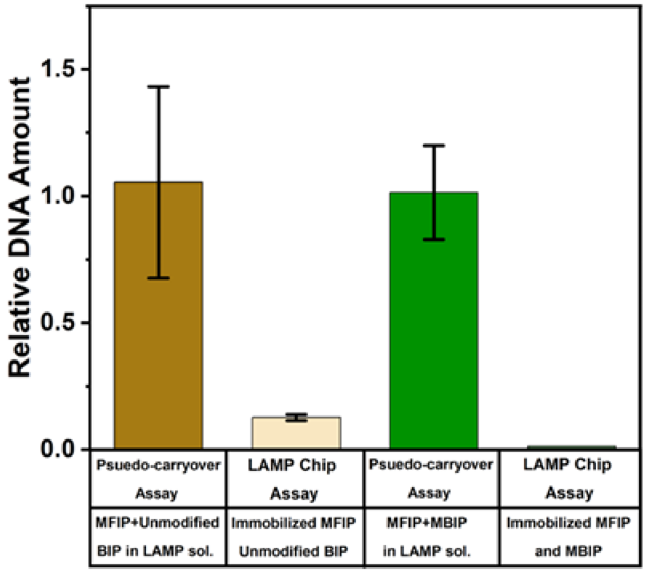

Figure S4. Real-time PCR of the pseudo-carryover and LAMP chip supernatants. The relative DNA amount was calculated using $2^{-\Delta\Delta Cq}$ method.

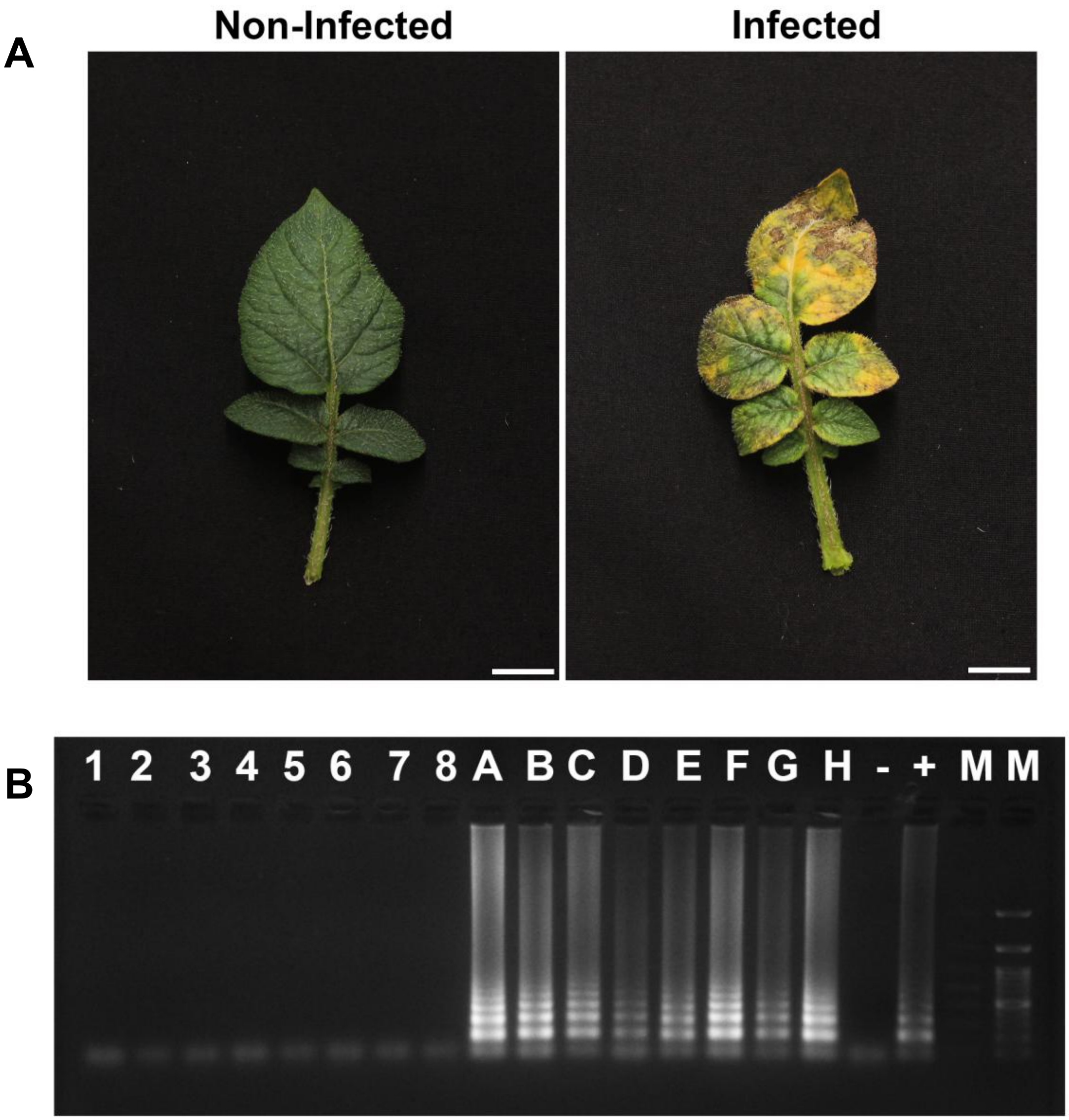

Figure S5. LAMP detection of *P. infestans* using tissue lysates from infected potato leaves. (A) Representative images of healthy and *P. infestans*-infected potato leaves. Scale bars represent 1 cm. (B) Conventional LAMP analysis of using crude tissue lysates from healthy (1-8) and *P. infestans*-infected (A-H) potato leaves. LAMP reactions with ddH2O (-) and *P. Infestans* DNA (+) as template were included as negative and positive controls. M, DNA ladder.

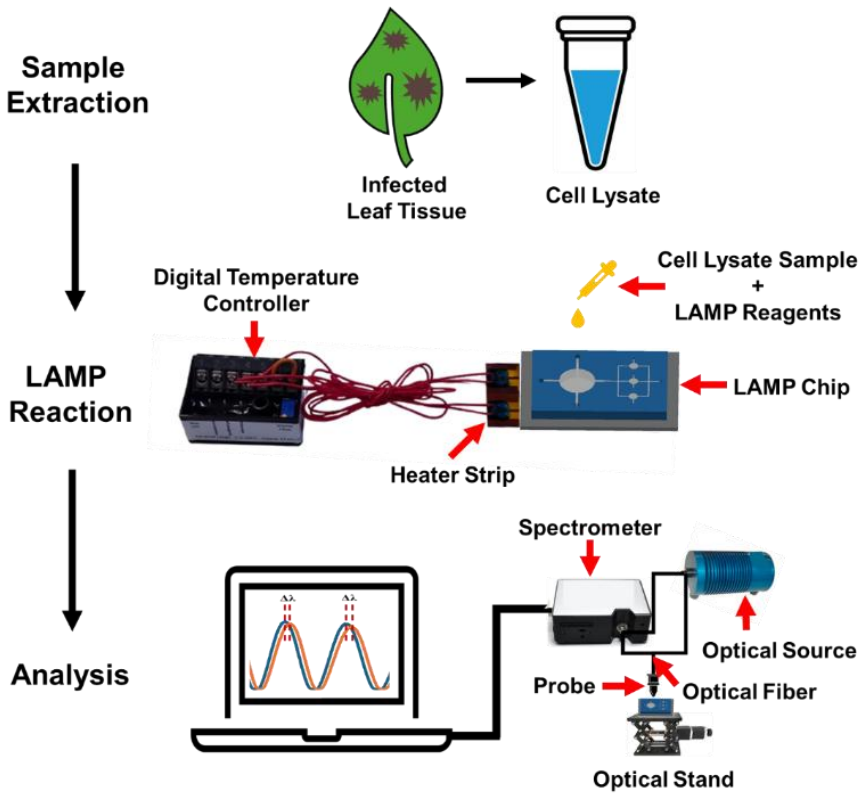

Figure S6: Field-ready workflow for rapid plant-pathogen detection using a LAMP-on-chip system.

Plant tissue lysate is extracted and loaded into the chip, which is then heated to 65 °C using a portable controller and flexible heating strip attached beneath the device. Following amplification, the chip is placed in a compact optical spectroscopy reader connected to a laptop for real-time data acquisition and analysis. All components fit within a small carry-case for point-of-care deployment.

References:

[1] Kong L., Wang H.-b., Wang S.-s., Xu P.-p., Zhang. R.-f., Dong S.-m. and Zheng X.-b., “Rapid detection of potato late blight using a loop-mediated isothermal amplification assay”, J. of Integrative Agriculture, 2020, 19(5): 1274–1282. https://doi.org/10.1016/S2095-3119(19)62816-9

[2] Ristaino J. B., Saville A. C., Paul R., Cooper D. C. and Wei Q., “Detection of Phytophthora infestans by Loop-Mediated Isothermal Amplification, Real-Time LAMP, and Droplet Digital PCR”, Plant disease, 2020, 104(3), 708-716. https://doi.org/10.1094/PDIS-06-19-1186-RE

[3] Hansen Z. R., Knaus B. J., Tabima J. F., Press C. M., Judelson H. S., Grünwald N. J. and Smart C. D., “Loop-mediated isothermal amplification for detection of the tomato and potato late blight pathogen, Phytophthora infestans”, J. Appl. Microbiology, 2016, 120 (4), 1010–1020, https://doi.org/10.1111/jam.13079

[4] Wharton S. P., Dangi S. and Woodhall J. W., “Development of an Innovative Loop-Mediated Isothermal Amplification (LAMP) Assay for the Rapid On-Site Detection of Phytophthora infestans”, Am. J. Potato Res., 2025, 102, 191–204. https://doi.org/10.1007/s12230-025-09986-6

[5] Mao S., Zhao J., Ding X., Vuong V. A., Song J. and Que L., “Integrated Sensing Chip for Ultrasensitive Label-Free Detection of the Products of Loop-Mediated Isothermal Amplification”, ACS Sens., 2023, 8, 2255−2262.
